# The pro-atherogenic response to disturbed blood flow is increased by a western diet, but not by old age

**DOI:** 10.1038/s41598-019-39466-x

**Published:** 2019-02-27

**Authors:** Ashley E. Walker, Sarah R. Breevoort, Jessica R. Durrant, Yu Liu, Daniel R. Machin, Parker S. Dobson, Elizabeth I. Nielson, Antonio J. Meza, Md. Torikul Islam, Anthony J. Donato, Lisa A. Lesniewski

**Affiliations:** 1 Department of Internal Medicine, University of Utah, Salt Lake City, Utah USA; 2 Department of Human Physiology, University of Oregon, Eugene, Oregon USA; 3 HistoTox Labs Inc., Boulder, Colorado USA; 4 Geriatrics Research Education and Clinical Center, Veteran’s Affairs Medical Center, Salt Lake City, Utah USA; 5 Department of Nutrition and Integrative Physiology, University of Utah, Salt Lake City, Utah USA

## Abstract

Atherogenic remodeling often occurs at arterial locations with disturbed blood flow (i.e., low or oscillatory) and both aging and western diet (WD) increase the likelihood for pro-atherogenic remodeling. However, it is unknown if old age and/or a WD modify the pro-atherogenic response to disturbed blood flow. We induced disturbed blood flow by partial carotid ligation (PCL) of the left carotid artery in young and old, normal chow (NC) or WD fed male B6D2F1 mice. Three weeks post-PCL, ligated carotid arteries had greater intima media thickness, neointima formation, and macrophage content compared with un-ligated arteries. WD led to greater remodeling and macrophage content in the ligated artery compared with NC mice, but these outcomes were similar between young and old mice. In contrast, nitrotyrosine content, a marker of oxidative stress, did not differ between WD and NC fed mice, but was greater in old compared with young mice in both ligated and un-ligated carotid arteries. In primary vascular smooth muscle cells, aging reduced proliferation, whereas conditioned media from fatty acid treated endothelial cells increased proliferation. Taken together, these findings suggest that the remodeling and pro-inflammatory response to disturbed blood flow is increased by WD, but is not increased by aging.

## Introduction

Advancing age and a diet high in saturated fat and/or sugar are risk factors for coronary heart disease and cardiovascular disease mortality^1–3^. At the same time, atherogenesis is most likely to occur at locations with disturbed blood flow (i.e., low or oscillatory shear stress), such as arterial branch points and curvatures^4^. However, it is unknown if old age and/or a western diet (WD, high saturated fat and sucrose) modify the pro-atherogenic response to disturbed blood flow.

The formation of an atherosclerotic plaque is a multi-stage process, with an early phase that includes an increase in the thickness of the medial layer of the artery partly due to vascular smooth muscle cell (VSMC) proliferation^5^. These proliferating VSMCs can also migrate toward the arterial lumen, leading to the formation of a neointima^5^. This proliferation is stimulated by growth factors, inflammatory cytokines, and reactive oxygen species produced by endothelial cells or immune cells within the arterial wall^6–9^. Low or disturbed shear stress across the endothelial surface leads to upregulation of genes related to inflammation, oxidative stress, and growth factors in endothelial cells and the release of these factors promotes VSMC proliferation^10–12^. Furthermore, an increase in inflammatory signals within the arterial wall leads to the recruitment of more immune cells that intensify the inflammatory environment^7^. However, it is unknown if aging and/or a WD affect the susceptibility of arteries to the pro-inflammatory, pro-oxidative and/or pro-VSMC proliferative response to disturbed blood flow. 

Most studies of aging and atherogenic remodeling examine arterial branch points and curvatures where disturbed blood flow occurs naturally, and thus, these studies are confounded by the cumulative lifelong exposure to this hemodynamic state. To overcome this limitation, we acutely induced disturbed blood flow *in vivo* by partial carotid ligation (PCL) in mice^13^. This method is preferred over other models of induced blood flow oscillation as it allows for continued, but limited, antegrade blood flow through the artery and does not denude the endothelium^13^. When performed in *ApoE*^*−/−*^ mice, PCL leads to the development of atherosclerotic plaques proximal to the site of ligation^13^. However, aged *ApoE*^*−/−*^ mice are also confounded by the lifelong exposure to altered lipid handling. Thus, we chose to examine wildtype mice for these studies. We first performed a time-course study to determine changes in hemodynamics and artery remodeling over time post-PCL in young mice. We then examined how the response to PCL differed with old age and WD.

We hypothesized that pro-atherogenic remodeling in response to PCL-induced disturbed blood flow would be greater with old age and WD alone, and further increased by the combination of the two. To test this hypothesis, we assessed pro-atherogenic remodeling by intima-media thickness (IMT) and neointima formation after PCL in young and old normal chow (NC) and WD fed mice. In addition, we hypothesized that markers of inflammation and oxidative stress would be greater after PCL with old age and WD, alone and in combination. We assessed inflammation by the presence of immune cells in the arterial wall and oxidative stress by nitrotyrosine content. As these studies focused on pro-atherogenic remodeling, rather than atherosclerotic plaque formation, wildtype mice were studied. To further examine the interaction of aging and fatty acids on VSMC proliferation, we performed studies on primary VSMCs collected from young and old mice. We examined the proliferative response of these young and old VSMCs to fatty acid treatment (palmitate) or to treatment with conditioned media from endothelial cells treated with palmitate.

## Results

### Time course for PCL response

**Figure 1:**
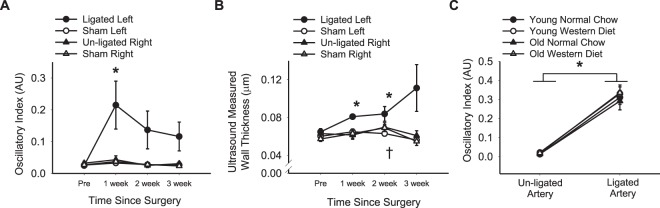
Time course for changes in oscillatory blood flow index and wall thickness after partial carotid ligation surgery in young mice and lack of differences between age and diet groups for the oscillatory blood flow response to partial carotid ligation. (**A**) Oscillatory blood flow index and (**B**) wall thickness measured by ultrasound in the left (ligated) and right (un-ligated) carotid arteries at pre-surgery and 1, 2, and 3 weeks post-surgery in young, normal chow fed B6D2F1 mice undergoing partial carotid ligation or sham surgery. “Un-ligated Right” is the contralateral (control) to the artery that underwent partial carotid ligation (“Ligated Left”). “Sham Right” is the contralateral (control) to the artery that underwent sham surgery (“Sham Left”). n = 4–5/group. ^*^p < 0.05 vs. pre-surgery ligated left. ^†^p < 0.05 vs. pre-surgery sham left. (**C**) Oscillatory blood flow index in the un-ligated and ligated carotid arteries after partial carotid ligation surgery in young and old normal chow and western diet fed B6D2F1 mice. n = 6–11/group. ^*^p < 0.05 RM-ANOVA un-ligated vs. ligated. Values are mean ± SEM.

PCL surgery was performed by ligating three of the four branches of the left carotid artery, allowing for continued antegrade blood flow through the superior thyroid artery. The right carotid artery was not ligated and served as a control. In young NC fed mice, the antegrade blood flow in the ligated (left) carotid artery decreased significantly by 1 week and remained reduced through measurements at 2 and 3 weeks post-PCL surgery (p < 0.05, Supplementary Fig. 1A). There were minimal, non-significant changes to retrograde blood flow in the ligated carotid artery post-PCL (p > 0.05, Supplementary Fig. 1B). The drastic reductions in antegrade blood flow with minimal changes to retrograde blood flow resulted in a greater oscillatory blood flow index for the ligated carotid artery post-PCL (p < 0.05, Fig. 1A) compared to pre-surgery. In the un-ligated (right) carotid artery, there were no changes in blood flow or oscillatory index at 1, 2, or 3 weeks post-PCL (p > 0.05, Fig. 1A, Supplementary Fig. 1A,B). In sham operated mice, no changes in blood flow or oscillatory index were observed in either left or right carotid arteries (p > 0.05, Fig. 1A, Supplementary Fig. 1A,B).

Ligated carotid artery wall thickness, measured by ultrasound, was increased at 1 and 2 weeks post-PCL surgery (p < 0.05), and tended to be greater at 3 weeks post-PCL (p = 0.07, Fig. 1B). By 3 weeks post-PCL ultrasound measurements of lumen diameter were significantly reduced in the ligated carotid artery (p = 0.03, Supplementary Fig. 1C). There were no changes in wall thickness or lumen diameter in the un-ligated carotid artery post-PCL or in either the left or right carotid arteries of sham operated mice (p > 0.05), except for a slightly greater wall thickness at week 2 in the right carotid artery of sham operated mice (p = 0.01, Fig. 1B, Supplementary Fig. 1C). Thus, PCL surgery induced increased oscillatory blood flow, increased wall thickness, and reduced diameter in the ligated artery, without effect on the un-ligated artery.

### Impact of age and WD on hemodynamic changes after PCL

In addition to young NC mice, we also performed PCL on old NC mice, as well as young and old mice fed a WD for 5 weeks pre-PCL and 3 weeks post-PCL. For all age and diet groups, PCL resulted in decreased antegrade blood flow and increased oscillatory blood flow index in the ligated carotid artery compared with the un-ligated carotid artery (p < 0.001, Table 1, Fig. 1C). Post-PCL antegrade and retrograde blood flow and oscillatory blood flow index did not differ between groups with the exception of antegrade blood flow in the un-ligated carotid artery, which was greater in old WD fed compared to all other groups (p < 0.05, Table 1, Fig. 1C). Thus, we conclude that the stimulus for pro-atherogenic remodeling (i.e., antegrade and oscillatory blood flow) was similar between groups for the ligated artery.

**Table 1:** Animal characteristics and hemodynamics after PCL surgery in ligated (left) and un-ligated (right) carotid arteries.

	Young Normal Chow	Young Western diet	Old Normal Chow	Old Western diet
Age (months)	6.5 ± 0.4	6.1 ± 0.2	29.0 ± 0.1^a,b^	29.7 ± 0.3^a,b^
Body mass (g)	32.7 ± 1.2	36.7 ± 1.7^a^	38.1 ± 1.7^a^	39.5 ± 1.5^a^
Ligated artery antegrade blood flow (ml/min)	0.32 ± 0.10	0.20 ± 0.03	0.22 ± 0.06	0.27 ± 0.10
Un-ligated artery antegrade blood flow (ml/min)	2.00 ± 0.16	2.38 ± 0.23	2.47 ± 0.28	4.11 ± 0.65 ^a,b,c^
Ligated artery retrograde blood flow (ml/min)	−0.11 ± 0.02	−0.11 ± 0.02	−0.08 ± 0.01	−0.12 ± 0.03
Un-ligated artery retrograde blood flow (ml/min)	−0.10 ± 0.02	−0.09 ± 0.01	−0.05 ± 0.00	−0.15 ± 0.05
Ligated artery lumen diameter (µm)	424 ± 18	438 ± 19	358 ± 12	411 ± 40
Un-ligated artery lumen diameter (µm)	447 ± 10	484 ± 11^a^	446 ± 13^b^	513 ± 16^a,c^

Data are mean ± SEM. ^a^p < 0.05 vs. young normal chow, ^b^p < 0.05 vs. young western diet, ^c^p < 0.05 vs. old normal chow.

### Arterial wall remodeling

**Figure 2:**
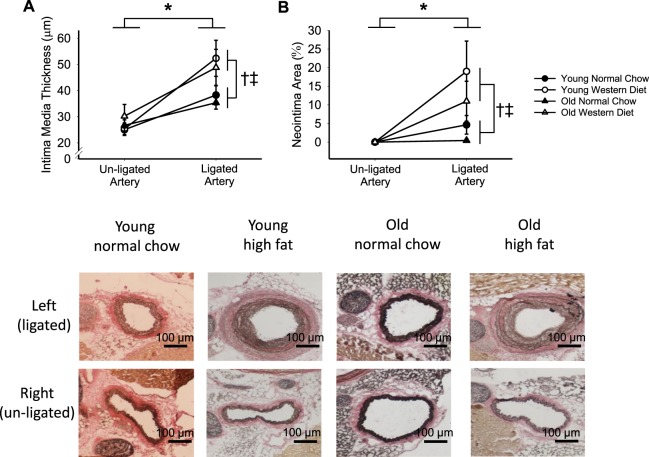
Western diet, but not old age, increases pro-atherogenic remodeling in response to partial carotid ligation. (**A**) Intima media thickness and (**B**) neointima area of the un-ligated and ligated carotid artery at 3-weeks after partial carotid ligation surgery in young and old, normal chow and western diet fed B6D2F1 mice. Neointima is calculated as the percent area within the internal elastic lamina that is composed of cells. Representative images are shown below. Elastic laminae stain dark brown-black with Verhoff-Van Gieson stain; scale bar = 100 µm. n = 7–11/group. ^*^p < 0.05 RM-ANOVA un-ligated vs. ligated. ^†^p < 0.05 ANOVA main effect normal chow vs. western diet, ^‡^p < 0.05 ANOVA interaction of ligation × diet.

Three weeks post-PCL, mice were euthanized and the carotid arteries dissected for histology and immunohistochemistry analyses. PCL induced remodeling of the artery, demonstrated by a ~70% greater IMT in the ligated compared with the un-ligated carotid artery in the combined group (RM-ANOVA: p < 0.001). Furthermore, WD fed mice had a greater increase in IMT in the ligated vs. un-ligated carotid arteries compared with NC fed mice (ligation × diet interaction: p = 0.02, main effect of diet: p = 0.04), but IMT was not different between young and old mice (main effect of age: p = 0.90, diet × age interaction: p = 0.97, Fig. 2A). Similar data trends were found when the data were expressed as the ratio of IMT to lumen diameter (data not shown).

**Figure 3:**
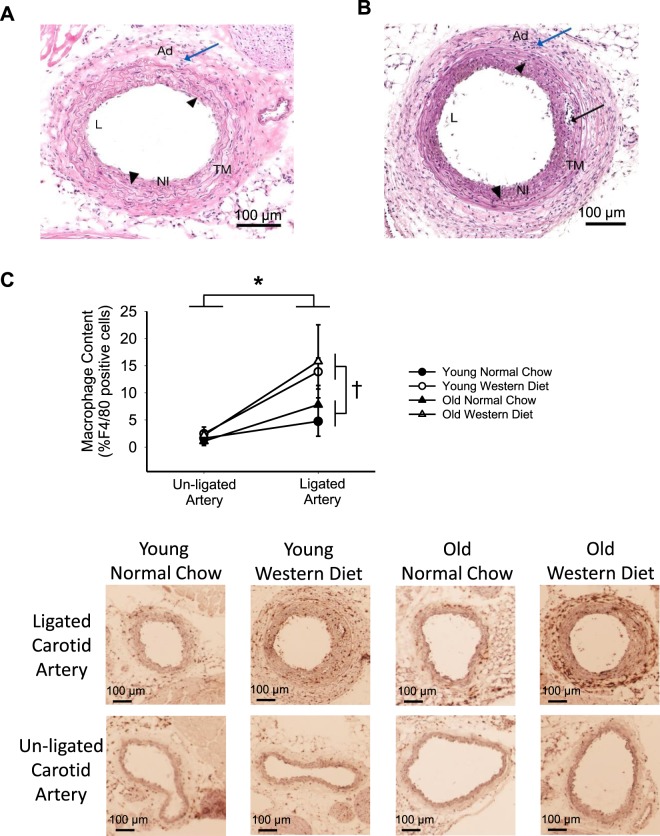
Arterial inflammation is greater with western diet, but not old age, in response to partial carotid ligation. In the young normal chow ligated carotid artery (**A**), infiltration of mononuclear inflammatory cells (blue arrow) in the adventitia is minimal and inflammation is absent in the neointima, within the internal elastic lamina (black arrowheads). In the western diet fed young ligated carotid artery (**B**), inflammatory cells (blue arrow) are dense within the adventitia, and neutrophils and mononuclear inflammatory cells (black arrow) also extend into the neointima. (**C**) The neointima is composed of vascular smooth muscle cells and extracellular matrix within the internal elastic lamina (black arrowheads). Macrophage content measured by the percent of F4/80 positive cells in the arterial wall in the ligated and un-ligated carotid arteries at 3-weeks after partial carotid ligation surgery in young and old, normal chow and western diet fed B6D2F1 mice n = 4–5/group. (**A**,**B**) are hematoxylin and eosin stain. Ad, adventitia; NI, neointima; TM, tunica muscularis; L, lumen. Below (**C**) are representative DAB stained F4/80, with hematoxylin stained nuclei. ^*^p < 0.05 RM-ANOVA un-ligated vs. ligated. ^†^p < 0.05 ANOVA main effect normal chow vs. western diet. Values are mean ± SEM.

While there was no neointima present in any un-ligated carotid artery, the neointima in the ligated carotid artery comprised on average ~10% of the area within the internal elastic lamina three weeks post-PCL (RM-ANOVA: p = 0.002). The increase in neointima area in ligated vs. un-ligated carotid arteries was greater in WD compared with NC fed mice (ligation x diet interaction: p = 0.02, main effect of diet: p = 0.02), but not different between young and old mice (main effect of age: p = 0.23, age × diet interaction: p = 0.68, Fig. 2B). Pathology assessment determined that the neointima was composed primarily of VSMCs and macrophages (see representative images in Fig. 3; Supplementary Fig. 2). There was no indication that lipids were present in the neointima (absence of oil red O straining, data not shown). Thus, PCL in wildtype mice induced remodeling characterized by thickening of the arterial wall and the formation of a neointima, but was without lipid accumulation as would be seen in an atheroma. WD, but not old age, led to a further increase in the arterial remodeling response to PCL, but still failed to lead to atheroma formation in this non-atheroprone wildtype mouse strain.

### Inflammation

By morphologic pathology, there was absent or minimal inflammation in the ligated carotid arteries of NC fed mice three weeks post-PCL. In contrast, ligated carotid arteries from WD fed mice had extensive adventitial and perivascular infiltration of mononuclear cells and neutrophils (inflammation) that extended into the tunica muscularis and intima in severe cases (Fig. 3A,B; Supplementary Fig. 2). Similarly, post-PCL macrophage content (F4/80 positive cells) was ~500% greater in the ligated compared with the un-ligated carotid artery in a combined group analysis (RM-ANOVA: p = 0.001). Furthermore, macrophage content was greater in WD fed compared with NC fed young mice (main effect of diet: p = 0.04, ligation x diet interaction: p = 0.06), but not different between young and old mice (main effect of age: p = 0.53, age x diet interaction: p = 0.88, Fig. 3C). In addition, there was no indication of T-cells (CD3+ immunohistochemistry) in the arterial wall or neointima of any group (data not shown). Therefore, PCL resulted in macrophage accumulation in the arterial wall, and this accumulation was greater with WD, but not old age.

### Oxidative stress

**Figure 4:**
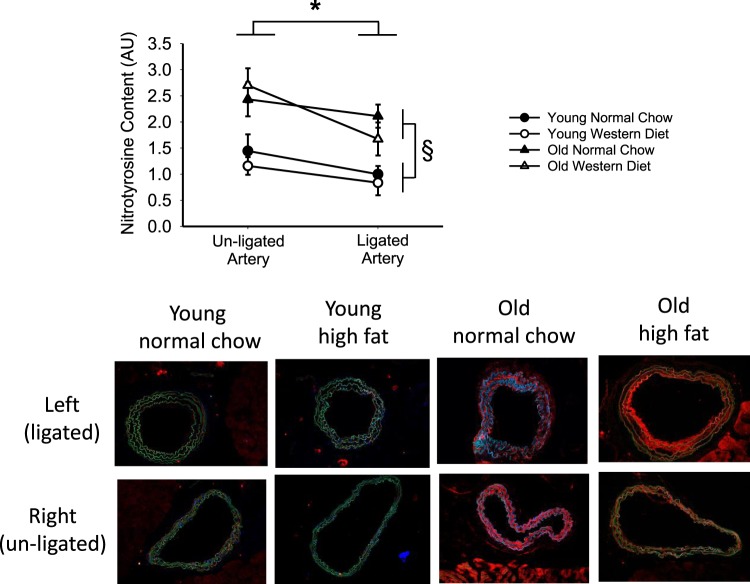
Arterial nitrotyrosine content is greater with old age, but not with western diet or partial carotid ligation. Nitrotyrosine content measured by immunofluorescence in the arterial wall in ligated and un-ligated carotid arteries 3-weeks after partial carotid ligation surgery in young and old, normal chow and western diet fed B6D2F1 mice. Representative images are below with nitrotyrosine in red, DAPI nuclei in blue, and autofluorescence of the elastic lamina in green. n = 7–9/group. Values are normalized to normal chow fed young left artery. ^*^p < 0.05 RM-ANOVA un-ligated vs. ligated. ^§^p < 0.05 ANOVA main effect young vs. old. Values are mean ± SEM.

In contrast to the above results, nitrotyrosine content, a marker of oxidative stress, was ~30% greater in the un-ligated artery compared with the ligated artery three weeks post-PCL in the combined group (RM-ANOVA: p = 0.003). Furthermore, nitrotyrosine content was greater in old compared with young (main effect of age: p < 0.001) mice, but there was no interaction of age and ligation status (ligation x age interaction: p = 0.38, Fig. 4). In addition, nitrotyrosine did not differ between groups for diet (main effect of diet: p = 0.49, age × diet interaction: p = 0.76, Fig. 4). Thus, nitrotyrosine content was not increased by PCL or WD, but was greater in old age.

### Effect of lifelong caloric restriction

In addition to NC and WD fed mice, we also studied a cohort of old mice who had undergone lifelong calorie restriction. IMT post-PCL did not differ between a cohort of old lifelong calorically restricted mice and old NC (ad libitum) fed mice in the ligated or un-ligated carotid artery (p > 0.05, Supplementary Fig. 3). As caloric restriction appeared to provide no benefit to old mice with respect to arterial remodeling, we did not perform any further analyses with the old caloric restriction group.

### VSMC proliferation response to fatty acid treatment

**Figure 5:**
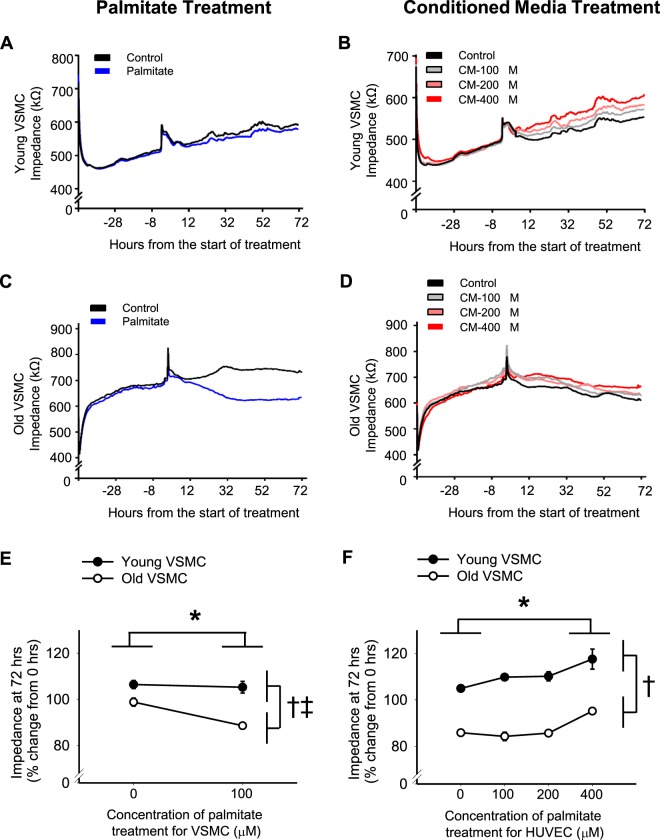
Young and old vascular smooth muscle cell proliferation in response to palmitate or conditioned media from palmitate treated endothelial cells. In vascular smooth muscle cells (VSMCs) collected from young (**A**,**B**) and old (**C**,**D**) mice, proliferation was measured by Electric Cell-substrate Impedance Sensing (ECIS), where an increase in impedance suggests proliferation of cells. 48 hours after seeding (time = 0 hr on figures), VSMCs were treated directly with 100 µM palmitate or vehicle control for 72 hr (**A**,**C**). An additional set of VSMCs were treated with conditioned media from cultured endothelial cells (HUVEC) exposed to palmitate at 100, 200, or 400 µM or with vehicle control (**B**,**D**) for 72 hr. (**A**–**D**) Are the impedance over time starting at seeding (−48 hr) and continuing through the treatment period (0–72 hr). (**E**,**F**) Are summary data for the change in impedance from the start of treatment (% change from 0 to 72 hours). n = 3/group. ^*^p < 0.05 ANOVA main effect of treatment. ^†^p < 0.05 ANOVA main effect of age, ^‡^p < 0.05 ANOVA interaction of treatment x age. Values are mean ± SEM.

As the neointima was primarily composed of VSMCs, we chose to further explore the interaction of age and fatty acid exposure on VSMC proliferation. To do so, we collected primary VSMCs from young or old mice and studied these cells *in vitro*. VSMC proliferation was measured in real time using Electric Cell-substrate Impedance Sensing (ECIS)^14^, where an increase in impedance suggests a proliferation of cells. VSMCs were seeded at 1.2 * 10^4^ cells/well in VSMC growth medium. Then, 48 hours after seeding, the cells were treated with palmitate, the most abundant dietary saturated fatty acid. At 48 hours after seeding (the start of treatment), there was no difference in the impedance (i.e., cell coverage) within age groups (p > 0.05, data not shown). Treatment with 100 µM palmitate resulted in reduced impedance after 72 hours in VSMCs, and this reduction was greater in cells from old compared with young mice (Fig. 5A,C,E; main effect of treatment: p = 0.006, main effect of age: p < 0.001, interaction of treatment x age: p = 0.021). The reduction in impedance indicates a decrease in cell number suggesting cell death due to palmitate toxicity, for which old VSMCs appear to be more sensitive. Higher concentrations of palmitate (200 or 400 µM) reduced impedance in both young and old VSMC cultures, suggesting that these higher concentrations of palmitate caused cell death in both groups (data not shown). Thus, direct treatment with palmitate does not stimulate proliferation of VSMCs and likely causes cell death in old VSMCs or at high palmitate concentrations.

Given the importance of endothelial cells as the barrier between the blood and VSMCs, as well as the importance for paracrine signaling between these cell types, we also treated endothelial cells with palmitate and then incubated VSMCs with conditioned media collected from these treated endothelial cells. For all conditioned media treatment conditions, VSMCs from old mice had a decrease in impedance during the treatment period, while VSMCs from young mice had an increase in impedance (Fig. 5B,D,F; main effect of age: p < 0.001). These findings suggest that young VSMCs had a proliferative response, whereas old VSMCs had a decrease in cell number due to cell death. Conditioned media from endothelial cells treated with 400 µM of palmitate increased impedance after 72 hours in VSMCs from both young and old mice (Fig. 5B,D,F; main effect of treatment: p < 0.001, post-hoc analyses: p < 0.05 400 µM vs. vehicle, 100 and 200 µM), suggesting that palmitate stimulates endothelial cells to release substances that promote VSMC proliferation (young VSMCs) and/or counteract cell death (old VSMCs). Furthermore, the interaction between age and treatment for these conditioned media experiments was not significant (age x treatment interaction: p = 0.098), indicating that the VSMC response to conditioned media from endothelial cells treated with palmitate does not change with age.

## Discussion

These are the first studies to examine the influence of aging and WD on the response to disturbed blood flow. We find that PCL resulted in greater oscillatory blood flow, IMT, neointima, and macrophage content in the ligated compared with un-ligated carotid arteries. We termed the greater IMT, neointima, and macrophage content as pro-atherogenic remodeling as these are some of the initial features of atherosclerotic development. However, the arteries did not develop atheroma as there was a lack of lipid accumulation. Importantly, we observed that a WD is associated with greater pro-atherogenic remodeling of the artery after PCL-induced disturbed blood flow. However, contrary to our hypothesis, aging does not appear to influence the remodeling response to PCL. Furthermore, oxidative stress appeared to increase with age in the carotid arteries, but was not increased by ligation. The pro-atherogenic remodeling in response to PCL was characterized by formation of a neointima primarily composed of VSMCs. In *in vitro* studies, both young and old VSMCs have a proliferative response to stimuli produced by endothelial cells in response to fatty acid exposure, however old VSMCs appear less proliferative than young VSMCs across all treatment conditions. These results demonstrate that a WD increases the amount of pro-atherogenic remodeling in response to disturbed blood flow. However, aging does not modulate pro-atherogenic remodeling in response to disturbed blood flow and may even lead to a decrease in VSMC proliferative capacity.

By utilizing ultrasound, we observed the time course for changes in blood flow, lumen diameter, and wall thickness in young NC fed wildtype mice after PCL surgery. As previously observed, we find antegrade blood flow is reduced at one-week post-PCL and remains low thereafter^13,15^. In addition, we find a slight, non-significant increase in retrograde blood flow at one-week post-PCL that returns to pre-PCL levels by two weeks. This normalization of retrograde blood flow is likely due to arterial remodeling, specifically the decrease in lumen size. Given these changes in antegrade and retrograde blood flow, the oscillatory blood flow index is greatest at one-week post-PCL. However, remodeling of the artery, indicated by increased wall thickness and decreased lumen diameter, occurs progressively over the 3 weeks post-PCL. Thus, arterial remodeling continues even after oscillatory blood flow begins to normalize. These findings suggest that the increase in oscillatory blood flow may be the initial trigger to start the remodeling process, but may not be needed to perpetuate this response.

When we examined the hemodynamic responses to PCL surgery in young and old, NC and WD fed mice, the only hemodynamic difference between groups was a greater antegrade blood flow in the un-ligated carotid artery of old WD mice compared with all other groups. Old WD mice also had a greater right carotid artery lumen diameter, similar to the greater carotid artery diameters seen in obese individuals^16^. However, the greater antegrade blood flow in the un-ligated artery of the old WD group is largely due to an augmented antegrade blood velocity. As we did not perform pre-surgery blood flow measures in the old WD group, we cannot determine if this is a difference present at baseline or a response to the surgery in the contralateral artery. Nevertheless, there were no hemodynamic differences between groups for the ligated carotid artery post-PCL. Therefore, the stimulus for pro-atherogenic remodeling was the same between groups, allowing us to isolate the effects of age and diet on the response to this stimulus.

Age is one of the greatest risk factors for atherosclerosis development^1^; although the reasons for this are not entirely clear. In *ApoE*^*−/−*^ and *Ldlr*^*−/−*^ models, old mice are more prone to atherosclerotic plaque development in areas with disturbed blood flow (e.g., the aortic arch) compared with young mice^17,18^. However, the limitation of these previous studies is that old mice are exposed to the pro-atherogenic stimuli (naturally occurring disturbed blood flow and genetic predisposition) over a lifetime. In the present study, we used wildtype mice that do not develop pro-atherogenic remodeling (neointima) naturally, allowing us to determine the response to acutely disturbed blood flow in old mice. In these wildtype mice, we observed increased IMT, neointima formation, and infiltration of immune cells post-PCL. We classify these changes as “pro-atherogenic” as they replicate many of the features of early atherosclerotic plaque development, but these are not atheroma as there is a lack lipid accumulation in the arterial wall. Similarly, previous studies have observed neointima formation in response to injury and PCL in wildtype mice^19,20^. It should also be noted that the WD in this study is designed to be obesogenic, unlike atherosclerotic diets that are high in cholesterol and cholate (Paigen diet)^21^. Therefore, our model allows us to isolate the effects of acute changes to disturbed blood flow and intake of saturated fat and sucrose, but is limited to only an early “pro-atherogenic” response and does not develop lipid-containing plaques. In addition, as we do not see differences in these pro-atherogenic characteristics between groups in the un-ligated carotid arteries, we conclude that WD alone did not induce pro-atherogenic remodeling in these wildtype mice, but rather altered the *response* to disturbed blood flow.

A key finding of this study is that age did not increase the propensity for pro-atherogenic remodeling in the face of disturbed blood flow. Furthermore, the combination of old age and WD did not increase pro-atherogenic remodeling beyond the response to WD alone, thus refuting our hypothesis. However, studies of human subjects indicate that oscillatory shear increases in conduit arteries with advancing age^22–24^. Thus, while aging does not appear to influence the response to an oscillatory blood flow stimulus, it may actually modify the stimulus itself, leading to a greater propensity for atherogenesis.

Rather than aging causing an increased neointima formation in response to disturbed blood flow as expected, we actually observed a trend for neointima formation to be less with old age in both NC and WD groups. As we identified the neointima to be primarily composed of VSMCs, this suggested an altered VSMC proliferation or survival with aging. In models of vascular injury, there are inconsistent results related to the age-related VSMC response, with some studies finding increased VSMC proliferation, while others find reduced proliferation with age^19,20,25–27^. To further explore the interaction of age and fatty acids (as in WD) on VSMC proliferation, we performed *in vitro* studies of primary VSMCs collected from young and old mice. We find that directly treating VSMCs with fatty acids appears to decrease cell numbers. Beyond the direct effects of fatty acids on VSMCs, dietary fatty acids may also change the phenotype of nearby cells to increase the release of factors activating VSMC proliferation. For example, endothelial cells are known to release growth factors and cytokines that increase VSMC proliferation^6–8,12^. Therefore, we treated young and old VSMCs with conditioned media from fatty acid-treated endothelial cells. We found that the conditioned media from fatty-acid treated endothelial cells stimulated proliferation in both young and old VSMCs, however the young VSMCs are more proliferative across all treatment conditions. These cell culture studies, however, do not model the effect of disturbed blood flow on endothelial cells, which is also known to result in the release of pro-proliferative VSMC stimuli^10–12^. Thus, further studies are needed to determine the VSMC proliferation response to PCL with old age and to examine the potential mechanism, particularly related to the responsiveness to pro-proliferative stimuli produced by endothelial cells.

Inflammation, specifically the recruitment of immune cells to the arterial wall, is a key step in atherosclerotic plaque development^7^. Previous studies examining acute increases in disturbed blood flow by PCL surgery in *ApoE*^*−/−*^ models have observed infiltration of macrophages and T-cells^28^. While we found an increase in macrophage recruitment after PCL in all age and diet groups, we did not observe the presence of T-cells in this wildtype model, suggesting that genetic alterations to lipid handling (i.e., *ApoE*^*−/−*^) may be needed for increased T-cell recruitment. Macrophage recruitment was greater with a WD, suggesting a synergistic interaction between fatty acids and disturbed blood flow that leads to signals for immune cell infiltration. However, old age does not lead to any further increases in immune cell recruitment in the presence of WD. Again, this is inconsistent with genetic atherosclerosis models, as aged *Ldlr*^*−/−*^ mice have a greater macrophage content in plaques compared to young^18^. Furthermore, our previous studies in B6D2F1 mice indicate that macrophages and T-cells increase with age in the aorta^29,30^, thus our present results are unexpected, as we do not observe greater immune cells in the un-ligated carotid arteries with age. This may result from intrinsic differences between the carotid arteries and the aorta or perhaps the smaller size of the carotid artery has limited our ability to detect immune cells. Nevertheless, it is clear that WD has a much greater influence on the pro-inflammatory response to disturbed blood flow than age.

Like inflammation, oxidative stress is a known cause of vascular dysfunction with advancing age^31^. We previously demonstrated that nitrotyrosine content, a marker of oxidative stress, increases with age in conduit arteries^32,33^. Previous studies of the response to PCL in *ApoE*^*−/−*^ mice indicate a key role of superoxide production by NADPH oxidase p47^phox^ in plaque development^13^. However, in the present study, greater nitrotyrosine content was observed with old age in both the ligated and un-ligated carotid arteries, and this was not influenced by WD. This suggests that the influence of aging overshadows the influences of disturbed blood flow or WD on oxidative stress. Thus, the patterns for group differences in oxidative stress are dissociated from those for pro-atherogenic remodeling post-PCL in this model. This indicates that oxidative stress may not be a contributing factor to pro-atherogenic remodeling in the absence of altered lipid handling (e.g., *ApoE*^*−/−*^), a possibility requiring further investigation.

In conclusion, the results of this study demonstrate that a WD, but not advanced age, increases the pro-atherogenic remodeling response to disturbed blood flow. These findings suggest that arterial locations with reduced antegrade blood flow or greater oscillatory blood flow are more susceptible to a pro-remodeling and pro-inflammatory phenotype in the presence of a WD. Furthermore, it appears that aging does not modulate arterial remodeling in response to disturbed blood flow, and thus aging likely increases atherosclerotic risk by other mechanisms.

## Methods

### Animals

Old B6D2F1 mice were obtained from the National Institute on Aging rodent colony maintained at Charles River Inc. and young B6D2F1 mice were obtained from the commercial colony maintained at Charles River Inc. All mice were housed in an animal care facility at the Salt Lake City VA Medical Center’s Animal Facility on a 12:12 light:dark cycle. Young and old male mice were fed a NC diet or a commercially available WD *ad libitum* for 5 weeks pre-PCL surgery and 3 weeks post-PCL surgery. NC diet was 14% fat, 54% carbohydrate, and 32% protein by kcal (EnvigoTeklad 8604). WD was 40% fat (41% saturated, 17% trans, 35% monounsaturated (cis) and 7% polyunsaturated (cis)), 41% carbohydrate (19% sucrose by weight) and 19% protein by kcal (Envigo Teklad 96132), as previously used^34^. Age and body mass for this main cohort are presented in Table 1. In addition, a cohort of lifelong caloric restriction mice was studied. These B6D2F1 mice were obtained from the National Institute on Aging rodent colony maintained at Charles River Inc. Calorie restriction was initiated at 14 weeks of age at ~10% below ad libitum, increased to ~25% restriction at 15 weeks and to ~40% restriction at 16 weeks, and maintained throughout the life of the animal. Caloric restriction mice were 29.1 ± 0.1 mo of age and weighed 28.4 ± 0.8 g. All animal procedures conformed to the *Guide for the Care and Use of Laboratory Animals* and were approved by Animal Care and Use Committee at the University of Utah and the Salt Lake City VA Medical Center.

### PCL surgery

PCL was performed as previously described^35^. Briefly, under isoflurane (1–2%) anesthesia, the left carotid artery was exposed by blunt dissection. 6–0 silk suture was used to ligate the external carotid distal to the superior thyroid artery and to ligate the internal carotid and occipital arteries. Thus, three of the four branches of the left carotid artery were ligated, allowing for continued antegrade blood flow through the superior thyroid artery. Sham surgeries involved blunt dissection to expose the left carotid artery, but no artery ligation.

### Ultrasound imaging and blood flow measurement

Imaging was performed using a Vevo 2100 ultrasound machine (Visual Sonics, FujiFilm, Toronto, ON, Canada) and a 32–56 MHz probe clamped in position. Mice were imaged under isoflurane (2%, 100% oxygen at 2 L/min flow rate) anesthesia. Antegrade and retrograde blood velocities were measured from pulse wave mode Doppler at the mid-point of the carotid artery with the insonation angle set at 65°. Wall thickness was measured at the mid-point of the carotid artery on the B-mode images. Lumen diameters were measured from B-mode images using edge detection software (Vascular Research Tools, Medical Imaging Applications LLC, Coraville, Iowa). Blood flow was calculated as BF = π * (d/2000)^2^ * v * 0.06, where BF is blood flow in ml/min, d is diameter in µm, and v is velocity in mm/s. Oscillatory index was calculated as OI = abs(BF_ret_)/(abs(BF_ant_) + abs(BF_ret_)), where OI is oscillatory index, abs(BF_ret_) is the absolute value of retrograde blood flow and abs(BF_ant_) is the absolute value of antegrade blood flow. Example B-mode ultrasound images and pulse wave Doppler are shown in (Supplementary Fig. 4).

### Histology and immunohistochemistry

Twenty-one days post-PCL, mice were sacrificed by thoracotomy under isoflurane anesthesia and perfused with saline through the left ventricle of the heart, with outflow through a cut in the inferior vena cava. The carotid arteries were collected *en bloc* with the trachea and esophagus, saved in optimal cutting temperature (OCT) solution, frozen, and sliced into 8 µm sections. Slides were stained separately with Verhoeff-Van Gieson (VVG) stain, hematoxylin and eosin (H&E), Masson’s trichrome, and Oil Red O by standard protocols^36^. A board (ACVP)-certified veterinary pathologist (JD) evaluated a subset of the VVG and H&E stained slides in a blinded manner using light microscopy (morphologic pathology). Using ImageJ, Masson’s trichrome images were used to measure intima media thickness and neointima formation. Neointima is reported as the percent area within the internal elastic lamina that is composed of cells. Macrophage and T-cell content was assessed by immunohistochemistry with primary antibodies for F4/80 (Abcam, Cambridge, MA) and CD3+ (Abcam, Cambridge, MA) respectively, using DAB and hematoxylin stained nuclei^29^. Using ImageJ, the number of nuclei and the number of F4/80 positive cells within the vascular wall were counted. For quantification of the oxidative stress marker, nitrotyrosine, sections were fixed, incubated with primary antibody for nitrotyrosine (Millipore, Burlington, MA) and AF647 secondary antibody, and were mounted with Prolong Gold with DAPI. Arteries were imaged with an Olympus Fluoview FV1000 (Tokyo, Japan) confocal microscope and to limit perivascular nitrotyrosine content inclusion, a projection of the Z slices only containing elastic lamina were used for analysis. Nitrotyrosine content was analyzed as the mean gray intensity within the artery wall using ImageJ software. For F4/80 and nitrotyrosine quantifications, one section on each slide did not receive primary antibody and final results were normalized to this no primary control^37^.

### Vascular smooth muscle cell isolation

Primary vascular smooth muscle cells were isolated from aorta of young (5 mo) and old (23 mo) C57BL6J mice, as previously described^38^. Briefly, the thoracic aorta was cleaned of surrounding fatty tissue, washed with PBS and incubated in an enzyme solution (HBSS buffer with 1 mg/ml collagenase II, 1 mg/ml soybean trypsin Inhibitor, 0.744 unit/ml elastase, and 1% penicillin/streptomycin) at 37 °C for 10 mins. After the incubation, the adventitia was removed, the aorta opened longitudinally, and the endothelial cells removed by gently scrapping with a sterilized cotton swab. The aorta was cut into small pieces and again incubated with enzyme solution at 37 °C for 1 hour. After the second incubation, the enzyme solution was neutralized by isolation medium (DMEM/F12 with 20%FBS and 2% Penicillin/streptomycin), the cells were pelleted, suspended in complete VSMC growth medium (DMEM/F12 with 10%FBS, 1% penicillin/streptomycin and 2 mM of L-glutamine) and plated. VSMCs were cultured in growth medium and passaged 3–5 times before the cell proliferation assay.

### Endothelial cell conditioned medium

Primary human umbilical vein endothelial cells (HUVEC, Lonza) were cultured in endothelial cell growth medium 2 (PromoCell). All experiments were performed using cells from passage 3 to 6. Sodium palmitate was dissolved in heated methanol (50 °C) to achieve the stock concentration as 100 mM. HUVECs were incubated with vehicle control or 100, 200, 400 µM of palmitate for 18 h as previously described^39^. This is a physiological concentration as fasting plasma palmitic acid in humans is ~300–500 µM^40,41^, while we measure total plasma free fatty acids to be ~700–900 µM in young/old, NC/WD fed mice^42^. After the incubation, the medium was changed to fresh VSMC growth medium. This VSMC growth medium was collected after 24 hours to be used in the cell proliferation assay.

### Cell proliferation assay

VSMC proliferation was measured in real time using an Electric Cell-substrate Impedance Sensing (ECIS) Zθ device (Applied Biophysics), following methods previously described^14^. VSMCs were grown on standard 96-well array (96W20idf PET). Briefly, arrays were coated with 10 μg/ml fibronectin (Alfa Aesar) for 30 minutes at 37 °C, washed with PBS, and then VSMCs were seeded at 1.2 * 10^4^ cells/well in VSMC growth medium. After 48 hours, the cells were treated with either conditioned medium (see above), sodium palmitate (100, 200, or 400 µM) or vehicle control for 3 days. Each treatment group was observed in triplicate. Data are presented as percentage changes in the impedance normalized to its initial value at 48 h.

### Data analysis

Statistical analyses were performed with IBM SPSS (version 20, Armonk, NY). Group differences for animal characteristics were assessed by one-way ANOVA with LSD post-hoc test when appropriate. All other group differences were assessed by a Repeated Measures (RM) ANOVA, with ligated vs. un-ligated artery as a within subjects contrast, and age and diet as between subjects contrasts. Values for nitrotyrosine content were normalized to the mean for the young normal chow fed left carotid artery. VSMC proliferation was analyzed by ANOVA (treatment x age), and post-hoc analyses by LSD where appropriate (100, 200, 400 µM conditioned media vs. control). Significance was set at p < 0.05. Values are presented as mean ± SEM.

## Supplementary information

Supplemental Figures

## Data Availability

The datasets generated during and/or analyzed during the current study are available from the corresponding author on reasonable request.

## References

[1] Lakatta EG, Levy D (2003). Arterial and cardiac aging: major shareholders in cardiovascular disease enterprises: Part I: aging arteries: a “set up” for vascular disease. Circulation.

[2] Clifton PM, Keogh JB (2017). A systematic review of the effect of dietary saturated and polyunsaturated fat on heart disease. Nutr Metab Cardiovasc Dis.

[3] Yang Q (2014). Added sugar intake and cardiovascular diseases mortality among US adults. JAMA Intern Med.

[4] VanderLaan PA, Reardon CA, Getz GS (2004). Site specificity of atherosclerosis: site-selective responses to atherosclerotic modulators. Arterioscler Thromb Vasc Biol.

[5] Dzau VJ, Braun-Dullaeus RC, Sedding DG (2002). Vascular proliferation and atherosclerosis: new perspectives and therapeutic strategies. Nat Med.

[6] Barillari G (2001). Inflammatory cytokines stimulate vascular smooth muscle cells locomotion and growth by enhancing alpha5beta1 integrin expression and function. Atherosclerosis.

[7] Ross R (1999). Atherosclerosis–an inflammatory disease. N Engl J Med.

[8] Galkina E, Ley K (2009). Immune and inflammatory mechanisms of atherosclerosis (*). Annu Rev Immunol.

[9] Jacot JG, Wong JY (2008). Endothelial injury induces vascular smooth muscle cell proliferation in highly localized regions of a direct contact co-culture system. Cell Biochem Biophys.

[10] Dardik A, Yamashita A, Aziz F, Asada H, Sumpio BE (2005). Shear stress-stimulated endothelial cells induce smooth muscle cell chemotaxis via platelet-derived growth factor-BB and interleukin-1alpha. J Vasc Surg.

[11] Hwang J (2003). Oscillatory shear stress stimulates endothelial production of O2- from p47phox-dependent NAD(P)H oxidases, leading to monocyte adhesion. J Biol Chem.

[12] Chatzizisis YS (2007). Role of endothelial shear stress in the natural history of coronary atherosclerosis and vascular remodeling: molecular, cellular, and vascular behavior. J Am Coll Cardiol.

[13] Nam D (2009). Partial carotid ligation is a model of acutely induced disturbed flow, leading to rapid endothelial dysfunction and atherosclerosis. Am J Physiol Heart Circ Physiol.

[14] Zhang X, Wang W, Li F, Voiculescu I (2017). Stretchable impedance sensor for mammalian cell proliferation measurements. Lab on a chip.

[15] Korshunov VA, Berk BC (2003). Flow-induced vascular remodeling in the mouse: a model for carotid intima-media thickening. Arterioscler Thromb Vasc Biol.

[16] Kappus RM (2014). Obesity and overweight associated with increased carotid diameter and decreased arterial function in young otherwise healthy men. Am J Hypertens.

[17] Reddick RL, Zhang SH, Maeda N (1994). Atherosclerosis in mice lacking apo E. Evaluation of lesional development and progression. Arterioscler Thromb.

[18] Du W (2016). Age-associated vascular inflammation promotes monocytosis during atherogenesis. Aging Cell.

[19] Wu X (2008). Ageing-exaggerated proliferation of vascular smooth muscle cells is related to attenuation of Jagged1 expression in endothelial cells. Cardiovasc Res.

[20] Vazquez-Padron RI (2004). Aging exacerbates neointimal formation, and increases proliferation and reduces susceptibility to apoptosis of vascular smooth muscle cells in mice. J Vasc Surg.

[21] Paigen B, Morrow A, Holmes PA, Mitchell D, Williams RA (1987). Quantitative assessment of atherosclerotic lesions in mice. Atherosclerosis.

[22] Padilla J (2011). Impact of aging on conduit artery retrograde and oscillatory shear at rest and during exercise: role of nitric oxide. Hypertension.

[23] Trinity JD (2014). Impact of age and body position on the contribution of nitric oxide to femoral artery shear rate: implications for atherosclerosis. Hypertension.

[24] Young CN, Deo SH, Padilla J, Laughlin MH, Fadel PJ (2010). Pro-atherogenic shear rate patterns in the femoral artery of healthy older adults. Atherosclerosis.

[25] Torella D (2004). Aging exacerbates negative remodeling and impairs endothelial regeneration after balloon injury. Am J Physiol Heart Circ Physiol.

[26] Khan SJ (2010). Stress-induced senescence exaggerates postinjury neointimal formation in the old vasculature. Am J Physiol Heart Circ Physiol.

[27] Stemerman MB (1982). Vascular smooth muscle cell growth kinetics *in vivo* in aged rats. Proc Natl Acad Sci USA.

[28] Li L, Chen W, Rezvan A, Jo H, Harrison DG (2011). Tetrahydrobiopterin deficiency and nitric oxide synthase uncoupling contribute to atherosclerosis induced by disturbed flow. Arterioscler Thromb Vasc Biol.

[29] Lesniewski LA (2011). Aerobic exercise reverses arterial inflammation with aging in mice. Am J Physiol Heart Circ Physiol.

[30] Trott DW (2016). Age-related arterial immune cell infiltration in mice is attenuated by caloric restriction or voluntary exercise. Exp Gerontol.

[31] Seals DR, Jablonski KL, Donato AJ (2011). Aging and vascular endothelial function in humans. Clin Sci (Lond).

[32] Lesniewski LA (2009). B6D2F1 Mice are a suitable model of oxidative stress-mediated impaired endothelium-dependent dilation with aging. J. Gerontol. A. Biol. Sci. Med. Sci..

[33] Durrant JR (2009). Voluntary wheel running restores endothelial function in conduit arteries of old mice: direct evidence for reduced oxidative stress, increased superoxide dismutase activity and down-regulation of NADPH oxidase. J Physiol.

[34] Lesniewski LA (2013). Aging compounds western diet-associated large artery endothelial dysfunction in mice: prevention by voluntary aerobic exercise. Exp Gerontol.

[35] Nam D (2010). A model of disturbed flow-induced atherosclerosis in mouse carotid artery by partial ligation and a simple method of RNA isolation from carotid endothelium. J Vis Exp.

[36] Donato AJ (2013). Life-long caloric restriction reduces oxidative stress and preserves nitric oxide bioavailability and function in arteries of old mice. Aging Cell.

[37] Walker AE (2015). Greater impairments in cerebral artery compared with skeletal muscle feed artery endothelial function in a mouse model of increased large artery stiffness. J Physiol.

[38] Cherepanova OA (2009). Oxidized phospholipids induce type VIII collagen expression and vascular smooth muscle cell migration. Circulation research.

[39] Li Y (2018). Novel role of PKR in palmitate-induced Sirt1 inactivation and endothelial cell senescence. American journal of physiology. Heart and circulatory physiology..

[40] Fraser DA, Thoen J, Rustan AC, Forre O, Kjeldsen-Kragh J (1999). Changes in plasma free fatty acid concentrations in rheumatoid arthritis patients during fasting and their effects upon T-lymphocyte proliferation. Rheumatology (Oxford).

[41] Ono-Moore KD (2016). Postprandial Inflammatory Responses and Free Fatty Acids in Plasma of Adults Who Consumed a Moderately High-Fat Breakfast with and without Blueberry Powder in a Randomized Placebo-Controlled Trial. J Nutr.

[42] Henson GD, Walker AE, Reihl KD, Donato AJ, Lesniewski LA (2014). Dichotomous mechanisms of aortic stiffening in high-fat diet fed young and old B6D2F1 mice. Physiol Rep.

