# Supplementary material for: The pro-atherogenic response to disturbed blood flow is increased by a western diet, but not by old age
Source: Sci Rep. 2019 Feb 27;9:2925. doi: 10.1038/s41598-019-39466-x (PMC6393500; doi:10.1038/s41598-019-39466-x)
Supplement: Supplementary file 1 — Supplemental Figures [file 41598_2019_39466_MOESM1_ESM.pdf]

Supplementary Figures

The pro-atherogenic response to disturbed blood flow is increased by a western diet, but not by old age

Ashley E Walker[1,2,*], Sarah R Breevoort[1], Jessica R Durrant[3], Yu Liu[1], Daniel R Machin[1,4], Parker S Dobson[1], Elizabeth I Nielson[1], Antonio J Meza[1], Md Torikul Islam[4], Anthony J Donato[1,4,5], Lisa A Lesniewski[1,4,5]

[1]Department of Internal Medicine, University of Utah, Salt Lake City, Utah; [2]Department of Human Physiology, University of Oregon, Eugene, Oregon; [3] HistoTox Labs Inc., Boulder, Colorado; [4]Geriatrics Research Education and Clinical Center, Veteran's Affairs Medical Center, Salt Lake City, Utah; [5]Department of Nutrition and Integrative Physiology, University of Utah, Salt Lake City, Utah

Supplemental Figure 1

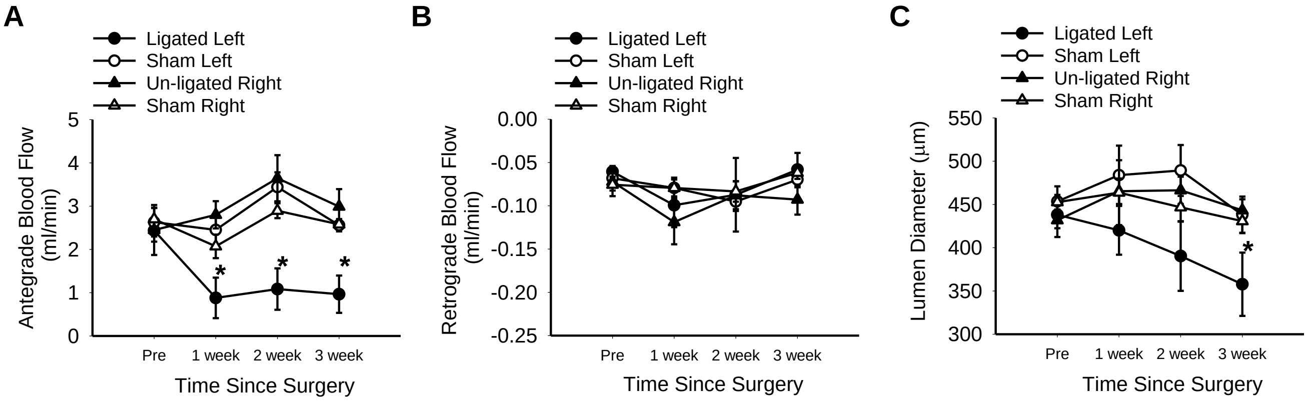

Supplemental Figure 1. Time course for changes in blood flow and lumen diameter after partial carotid ligation. (A) Antegrade blood flow, (B) retrograde blood flow, and (C) lumen diameter measured by ultrasound in the ligated (left) and un-ligated (right) carotid arteries at pre-surgery and 1, 2, and 3 weeks post-surgery. Measurements are made in young, normal chow fed B6D2F1 mice undergoing PCL or sham surgery. n=4-5/group. *$p<0.05$ vs. pre-surgery ligated left. Values are mean±SEM.

Supplemental Figure 2

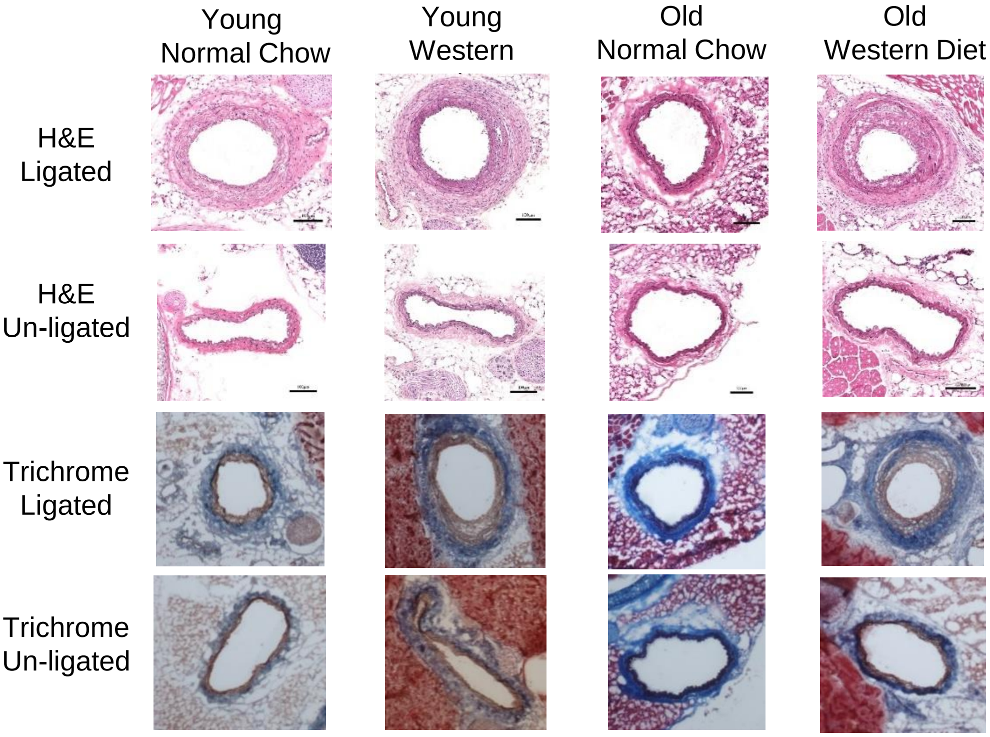

Supplemental Figure 2. Representative images for hematoxylin and eosin (H&E) and Masson's trichrome stain. Staining for ligated (left) and un-ligated (right) carotid arteries 3 weeks post-partial carotid ligation in young and old normal chow and western diet fed B6D2F1 mice.

Supplemental Figure 3

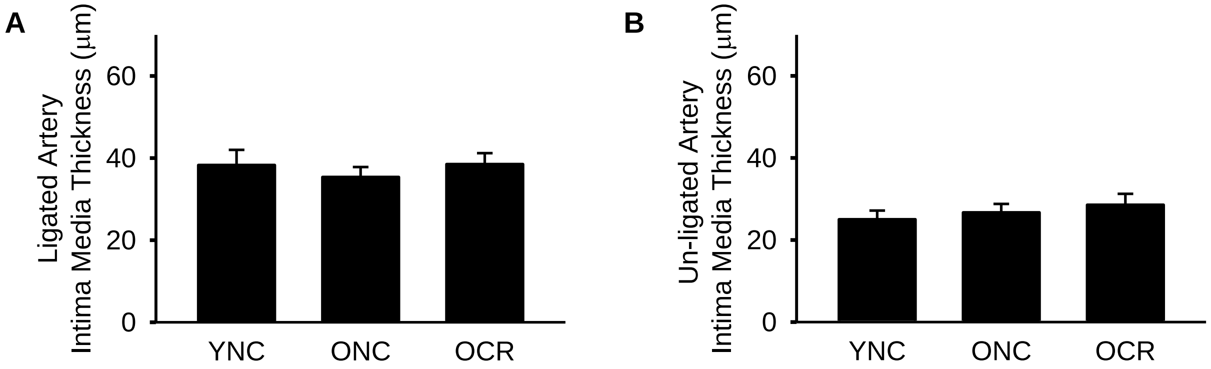

Supplemental Figure 3. Lifelong caloric restriction did not result in a different response to partial carotid ligation compared with *ad libitum* normal chow feeding. Intima media thickness in (A) the ligated (left) carotid artery and (B) the un-ligated (right) carotid artery in young normal chow (YNC) and old normal chow ONC and old lifelong caloric restriction (OCR) B6D2F1 mice. n=7-11/group. Values are mean±SEM.

Supplemental Figure 4

Ligated (left) carotid artery post-PCL surgery | **Un-ligated (right) carotid artery post-PCL surgery**

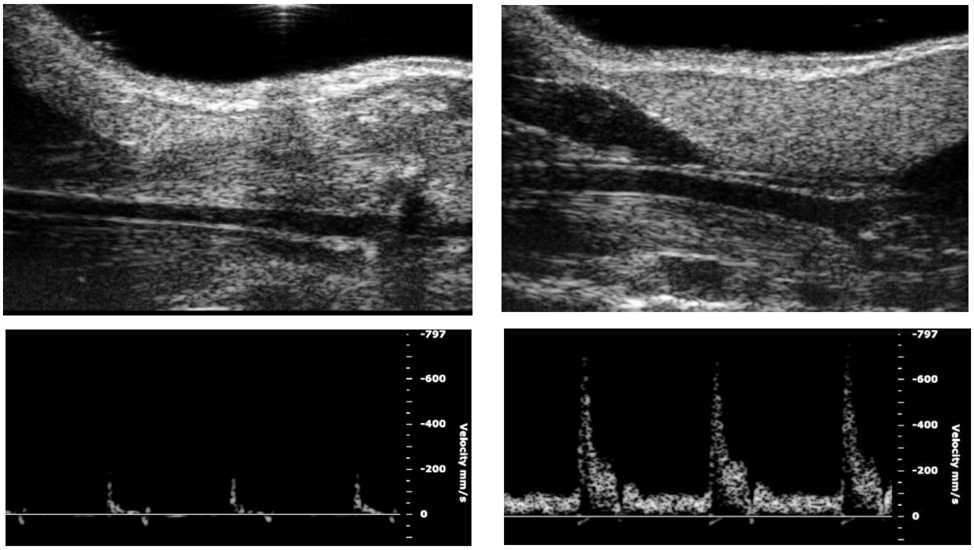

Supplemental Figure 4. Example B-mode ultrasound and Doppler pulse wave of the carotid artery. Ultrasound images of the carotid arteries from a mouse after partial carotid ligation (PCL) surgery to the left carotid artery. A thicker arterial wall and smaller lumen diameter can be noted in the ligated carotid artery compared to the un-ligated carotid artery. Below are the Doppler blood velocities demonstrating reduced antegrade blood velocity in the ligated carotid artery after PCL surgery.
